# Link of dysfunctional attitudes with the negative self-model

**DOI:** 10.1186/s12991-016-0098-y

**Published:** 2016-03-15

**Authors:** Koichi Otani, Akihito Suzuki, Yoshihiko Matsumoto, Masanori Enokido

**Affiliations:** Department of Psychiatry, Yamagata University School of Medicine, 2-2-2 Iidanishi, Yamagata, 990-9585 Japan

**Keywords:** Dysfunctional attitude, Achievement/dependency/self-control, Self-model/other-model, DAS-24, RSQ

## Abstract

**Background:**

Beck’s cognitive theory postulates that dysfunctional attitudes predisposing to depression are formed by early negative experiences. Meanwhile, Bowlby’s attachment theory contends that distorted working models built through insecure attachment relationships lead to various psychopathologies such as depression. The present study examined the correlations of dysfunctional attitudes about achievement, dependency, and self-control with working models of the self and other, and tried to promote understanding of those dysfunctional attitudes from an attachment perspective.

**Methods:**

The subjects were 591 Japanese healthy volunteers. Dysfunctional attitudes about achievement, dependency, and self-control were evaluated by the corresponding subscales of the 24-item Dysfunctional Attitude Scale, and working models of the self and other were assessed by the relationship scales questionnaire.

**Results:**

The scores of the achievement (*β* = −0.26, *P* < 0.001), dependency (*β* = −0.41, *P* < 0.001), and self-control (*β* = −0.14, *P* < 0.01) subscales had negative correlations with the self-model score, suggesting the connections of all clusters of dysfunctional attitudes with the negative self-model. The score of the dependency subscale (*β* = 0.21, *P* < 0.001) had a positive correlation with the other-model score, suggesting the connection of this cluster of dysfunctional attitudes with the positive other-model. Meanwhile, the scores of the achievement (*β* = −0.17, *P* < 0.001) and self-control (*β* = −0.13, *P* < 0.01) subscales had negative correlations with the other-model score, suggesting the connections of these clusters of dysfunctional attitudes with the negative other-model.

**Conclusion:**

The present study suggests that dysfunctional attitudes as a whole are linked with the negative self-model built through negative attachment experiences, while the content specificity of each cluster is related to the differential correlations with the other-model.

**Electronic supplementary material:**

The online version of this article (doi:10.1186/s12991-016-0098-y) contains supplementary material, which is available to authorized users.

## Background

Beck’s cognitive theory postulates that maladaptive beliefs about one’s self and personal world formed at an early stage of development predispose to depression [1, 2]. Once these maladaptive self-schemas are activated by life stressors, cognitive errors and negative automatic thoughts ensue, resulting in negative interpretations and self-evaluations that characterize clinical depression. Weissman and Beck [3] developed the Dysfunctional Attitude Scale (DAS) to evaluate these maladaptive self-schemas. Subsequently, Power et al. [4] factor analyzed the DAS items and obtained three factors which they termed achievement, dependency, and self-control. Based on this result, they developed a 24-item version of the DAS (DAS-24) with corresponding three subscales. The achievement subscale contains items about achievement and failure, the dependency subscale contains those about dependency and approval, and the self-control subscale contains those about necessity for self-control. Power et al. [4] propose that this subscaled version of the DAS is more useful than global measures such as the DAS for the individualized assessment of cognitive vulnerability, since a depression-prone individual may develop dysfunctional attitudes not in all areas but in limited area(s) of life on which that individual places a high value.

According to Bowlby’s attachment theory [5, 6], the crucial roles of parents are, first, to respond to a child’s desire for care and, second, to encourage a child to explore the world, developing secure attachment in the child. In contrast, lack of care and/or overprotection of parents create insecure attachment in a child. These attachment relationships are internalized to form working models of the self and other. The positive self-model is characterized by the image of the self as effective and worthy, while the negative self-model is characterized by the image of the self as helpless ad unworthy. The positive other-model is characterized by the image of the other as reliable and supportive, while the negative other-model is characterized by the image of the other as unreliable and rejecting. Bartholomew [7] postulates that negativity of the self-model is externalized as dependency, i.e., need for others’ approval to maintain a positive self-regard, while negativity of the other-model is externalized as avoidance, i.e., avoidance of closeness to protect against disappointment.

There are some similarities between dysfunctional attitudes [1, 2] and distorted working models [5, 6], i.e., the tendency to persist relatively unchanged throughout life, the routine influence on thought, feeling, and behavior, and the predisposition to psychopathologies such as depression. Furthermore, Beck and collaborators [1, 2] presume that dysfunctional attitudes are formed by negative childhood experiences, especially with parents. In fact, the study by Randolf and Dykman [8] has suggested that low care and overprotection by parents increase dysfunctional attitudes. Also, our previous study [9] has suggested that parental overprotection engenders dysfunctional attitudes about achievement and dependency. These discussions point to the possibility that dysfunctional attitudes are linked with distorted working models originating from negative attachment experiences. However, as far as we know no study has examined the association between dysfunctional attitudes and the two working models. Therefore, the purposes of the present study were to examine the relationships of dysfunctional attitudes about achievement, dependency, and self-control with working models of the self and other, and to promote understanding of those dysfunctional attitudes from an attachment perspective. Specifically, we expected that the present study would clarify the close relation of dysfunctional attitudes with negative working models, especially that of the self, and shed some light on the formation mechanism of each cluster of dysfunctional attitudes.

## Methods

Originally, 632 physically healthy Japanese were recruited from medical students and hospital staffs in Yamagata Prefecture. The psychiatric screening consisted of interviews by well-trained psychiatrists and a questionnaire on current or past psychiatric treatment and diagnosis. For the psychiatric interview, six items were selected from the Structured Clinical Interview for DSM-IV Axis I Disorders [10], i.e., A1 for major depressive episode, A16 for manic episode, B1 for delusions, B6 for hallucinations, E2 for alcohol abuse, and F68 for anxiety disorders. Out of the 632 cases, 15 had current or past psychiatric disorders and 26 had missing data. These 41 cases were excluded, and the remaining 591 cases were used for analyses. The study protocol was approved by the Ethics Committee of Yamagata University School of Medicine, and all subjects provided written informed consent to participate.

Dysfunctional attitudes about achievement, dependency, and self-control were evaluated by the corresponding subscales of the Japanese version of the DAS-24 [11] (Additional file 1: Appendix 1), which has high reliability and validity. The achievement subscale consists of 8 items, e.g., “If I fail partly, it is as bad as being a complete failure.” The dependency subscale consists of eight items, e.g., “I am nothing if a person I love doesn’t love me.” The self-control subscale consists of eight items, e.g., “A person should do well at everything he undertakes.” Respondents rate the degree to which they match each phrase on a 1- to 7-point scale, where 1 is “totally disagree” and 7 is “totally agree.” In the present sample, Cronbach’s alphas for the achievement, dependency, and self-control subscales were 0.78, 0.75, and 0.60, respectively.

Working models of the self and other were assessed by the method of Griffin and Bartholomew [12]. Firstly, the secure, dismissing, preoccupied and fearful attachment styles derived from combinations of positivity or negativity of the two working models were assessed by the corresponding four subscales of the Japanese version [13] (Additional file 2: Appendix 2) of the relationship scales questionnaire [12]. Reliability and validity of this Japanese version have been confirmed [13]. The secure subscale consists of five items, e.g., “I find it easy to get emotionally close to others.” The dismissing subscale consists of five items, e.g., “I am comfortable without close emotional relationships.” The preoccupied subscale consists of four items, e.g., “I want to be completely emotionally intimate with others.” The fearful subscale consists of four items, e.g., “I worry that I will be hurt if I allow myself to become too close to others.” Respondents rate the degree to which they match each phrase on a 5-point scale ranging from “not at all like me” to “very like me.” In the present sample, Cronbach’s alphas for the secure, dismissing, preoccupied, and fearful subscales were 0.53, 0.55, 0.62, and 0.67, respectively. The self-model score is obtained by summing the ratings of the two styles with positive self-models (secure and dismissing) and subtracting the ratings of the two styles with negative self-models (preoccupied and fearful). The other-model score is obtained by summing the ratings of the two styles with positive other-models (secure and preoccupied) and subtracting the ratings of the two styles with negative other-models (dismissing and fearful).

Statistical analyses were conducted by the forced entry multiple regression analysis using SPSS 14.0 J for Windows (SPSS Japan Inc, Tokyo, Japan). The dependent variables were the achievement, dependency, and self-control subscale scores, and the independent variables were the self-model and other-model scores. A *P* < 0.05 was considered statistically significant.

## Results

Of the 591 subjects, 435 were males and 156 were females. The mean ± SD of age was 28.6 ± 8.4 years.

Table 1 shows the DAS-24 and RSQ scores of the subjects.

**Table 1:** DAS-24 and RSQ scores of the subjects

DAS-24	
Achievement	27.1 ± 8.1
Dependency	31.9 ± 7.3
Self-control	30.0 ± 6.2
RSQ	
Secure	17.0 ± 3.0
Dismissing	12.2 ± 2.9
Preoccupied	10.4 ± 2.4
Fearful	10.4 ± 3.0
Self-model	8.4 ± 6.3
Other-model	4.8 ± 6.8

Figures in the table show mean ± SD

*DAS-24* 24-item dysfunctional attitude scale, *RSQ* relationship scales questionnaire

Table 2 shows the results of multiple regression analyses of the DAS-24 subscale scores with the self-model and other-model scores. The scores of the achievement (*P* < 0.001), dependency (*P* < 0.001), and self-control (*P* < 0.01) subscales had negative correlations with the self-model score. The score of the dependency subscale (*P* < 0.001) had a positive correlation, while the scores of the achievement (*P* < 0.001) and self-control (*P* < 0.01) subscales had negative correlations, with the other-model score.

**Table 2:** Multiple regression analyses of DAS-24 subscale scores with the self- and other-model scores

	Achievement	Dependency	Self-control
Self-model	*β* = −0.260	*β* = −0.409	*β* = −0.142
	*t* = −6.390**	*t* = −10.254**	*t* = −3.344*
Other-model	*β* = −0.168	*β* = 0.209	*β* = −0.125
	*t* = −4.125**	*t* = 5.229**	*t* = −2.951*
Correlation coefficient	*R* = 0.351	*R* = 0.396	*R* = 0.217
	*F* = 41.311**	*F* = 54.800**	*F* = 14.490**

*DAS-24* 24-item dysfunctional attitude scale

* *P* < 0.01, ** *P* < 0.001

## Discussion

In the present study, all subscale scores of the DAS-24 had negative correlations with the self-model score. This result suggests that dysfunctional attitudes as a whole are linked with the negative self-model, i.e., the image of the self as helpless and unworthy [5–7]. Therefore, the present study using the attachment framework confirms and supports the key concept of cognitive theory of depression that helplessness and unworthiness are the major core beliefs organized in depressive self-schemas [1, 2]. The present result is also in line with our previous result [14] that dysfunctional attitudes measured by the DAS-24 were correlated with low scores of the self-directedness dimension of the temperament and character inventory (TCI) [15], which is the concept of the self as an autonomous individual.

The three subscales of the DAS-24 had differential correlations with the other-model, in contrast to the uniform correlation with the self-model. The dependency subscale had a correlation with the positive other-model, i.e., the image of the other as reliable and supportive, while the achievement and self-control subscales had correlations with the negative other-model, i.e., the image of the other as unreliable and rejecting. These differential correlations with the other-model may be related to the content specificity of each cluster of dysfunctional attitudes. That is, to maintain a positive self-regard in the face of the negative core beliefs, a person with a positive other-image may tend to develop conditional rules, imperative beliefs, and compensatory beliefs on interpersonal relationships, while that with a negative other-image may tend to develop those rules or beliefs on impersonal aspects of life such as individualistic achievement and necessity for self-control. Incidentally, the correlations of the three subscales with the other-model are also reflected in the results of our previous study using the TCI [14], i.e., the dependency subscale was connected with high reward dependence characterized by intimacy and dependency [15], while the achievement and self-control subscales were connected with low cooperativeness characterized by social disinterest and self-absorption [15].

The correlations of the three clusters of dysfunctional attitudes with distorted working models suggest that those dysfunctional attitudes are associated with negative attachment relationships. We previously examined the effects of parental care and protection assessed by the parental bonding instrument [16] on dysfunctional attitudes measured by the DAS-24, and found that parental overprotection increased dysfunctional attitudes about achievement and dependency [9]. Therefore, it is speculated that negative attachment experiences with parents are implicated in the formations of distorted working models, especially the negative self-model, and dysfunctional attitudes, especially those about achievement and dependency. On the other hand, dysfunctional attitudes about self-control were not affected by parental rearing in that study [9], but they still are linked with negative working models of the self and other as presented here. Therefore, other attachment experiences such as those with siblings, peers, and partners may play a more important role in the formation of dysfunctional attitudes about self-control.

Clinical implications of the present results for the clinical practice of depression are as follows. Firstly, as depressed patients with dysfunctional attitudes are likely to have negative attachment experiences in early life, the therapist may have to deal with them at least partly, though focusing on here-and-now problems is the principle of cognitive therapy [17]. Secondly, the therapist may have to cope with distinctive interpersonal behaviors according to the types of dysfunctional attitudes harbored by the patients. The patients with dysfunctional attitudes about dependency accompanied by the positive other-model are likely to reach out to others for fulfillment of dependency needs [7]. Therefore, the setting of limits or boundaries may be necessary for them. Meanwhile, the patients with dysfunctional attitudes about achievement and self-control accompanied by the negative other-model are likely to avoid close involvement with others for fear of rejection [7]. Therefore, considerable effort may be required to form therapeutic alliance with them.

There are two possible limitations in the present study. Firstly, the psychiatric screening conducted, i.e., a brief interview and a questionnaire on psychiatric disorders, might not be sufficient, and therefore, the present results may be influenced by psychiatric symptoms of the subjects with those disorders. Secondly, all subjects were Japanese medical students or hospital staffs, i.e., relatively young Japanese with high educational background, and therefore, the present results may not be extrapolated directly to other ethnic groups or general populations.

## Conclusion

The present study suggests that dysfunctional attitudes as a whole are linked with the negative self-model built through negative attachment experiences, while the content specificity of each cluster is related to the differential correlations with the other-model.

## Additional files

10.1186/s12991-016-0098-y. 24-item Dysfunctional Attitude Scale 日本語版.
10.1186/s12991-016-0098-y. Relationship scales questionnaire 日本語版.
